# Supplementary material for: Distinct Roles for RAB10 and RAB29 in Pathogenic LRRK2-Mediated Endolysosomal Trafficking Alterations
Source: Cells. 2020 Jul 17;9(7):1719. doi: 10.3390/cells9071719 (PMC7407826; doi:10.3390/cells9071719)
Supplement: Supplementary file 1 [file cells-09-01719-s001.pdf]

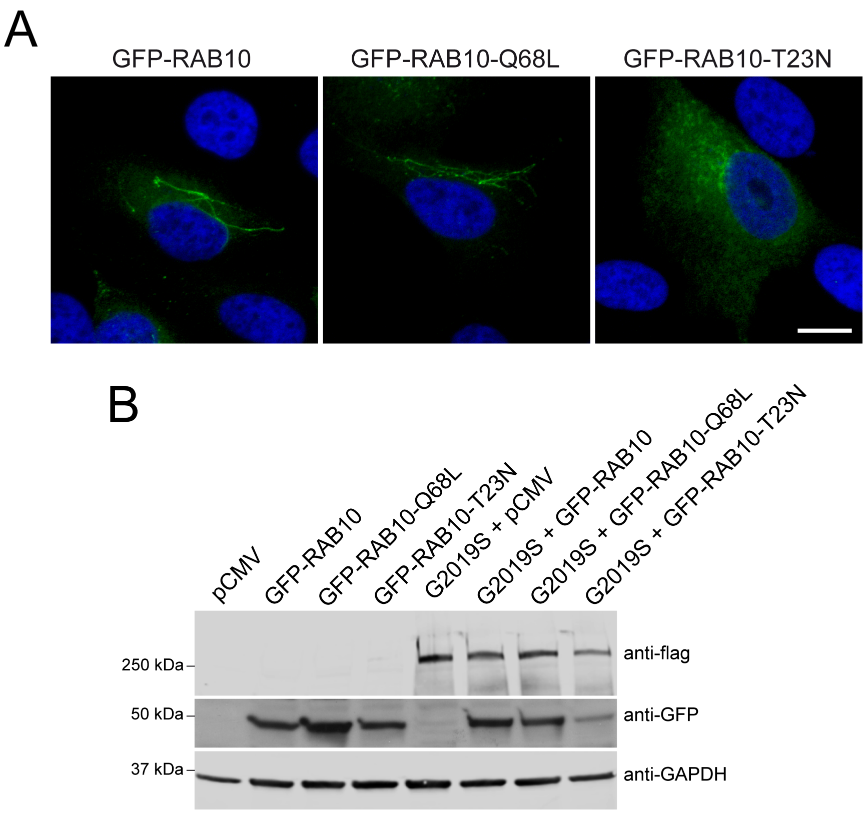

Supplementary Figure 1. Subcellular localization and expression levels of RAB10 variants. (**A**) HeLa cells were transfected with the different GFP-tagged RAB10 constructs as indicated, and fixed but only briefly permeabilized before mounting with DAPI to maintain proper membrane association. Scale bar, 10 μm. (**B**) HeLa cells were transfected with empty pCMV vector, with GFP-tagged RAB10 constructs, or cotransfected with flag-G2019S LRRK2 along with GFP or GFP-tagged RAB10 constructs as indicated, and cell extracts (30 μg) analyzed by Western blotting for flag, GFP, and GAPDH as loading control.

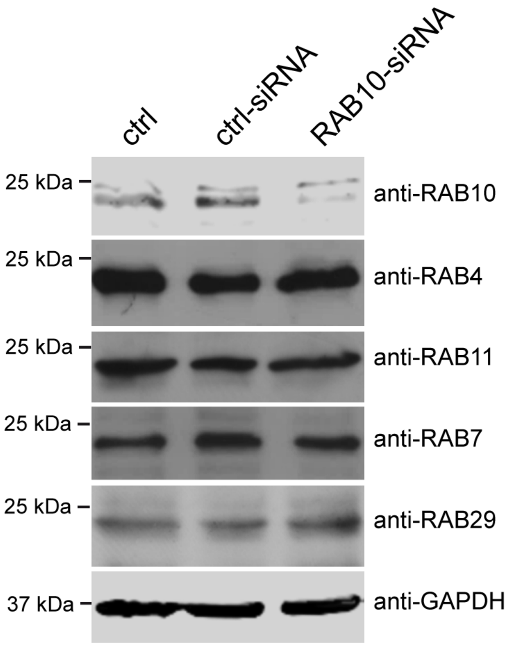

Supplementary Figure 2. Specificity of RAB10 knockdown. HeLa cells were treated with either ctrl-siRNA or RAB10-siRNA, and extracts subjected to Western blotting against various RAB proteins as indicated, with GAPDH as loading control.

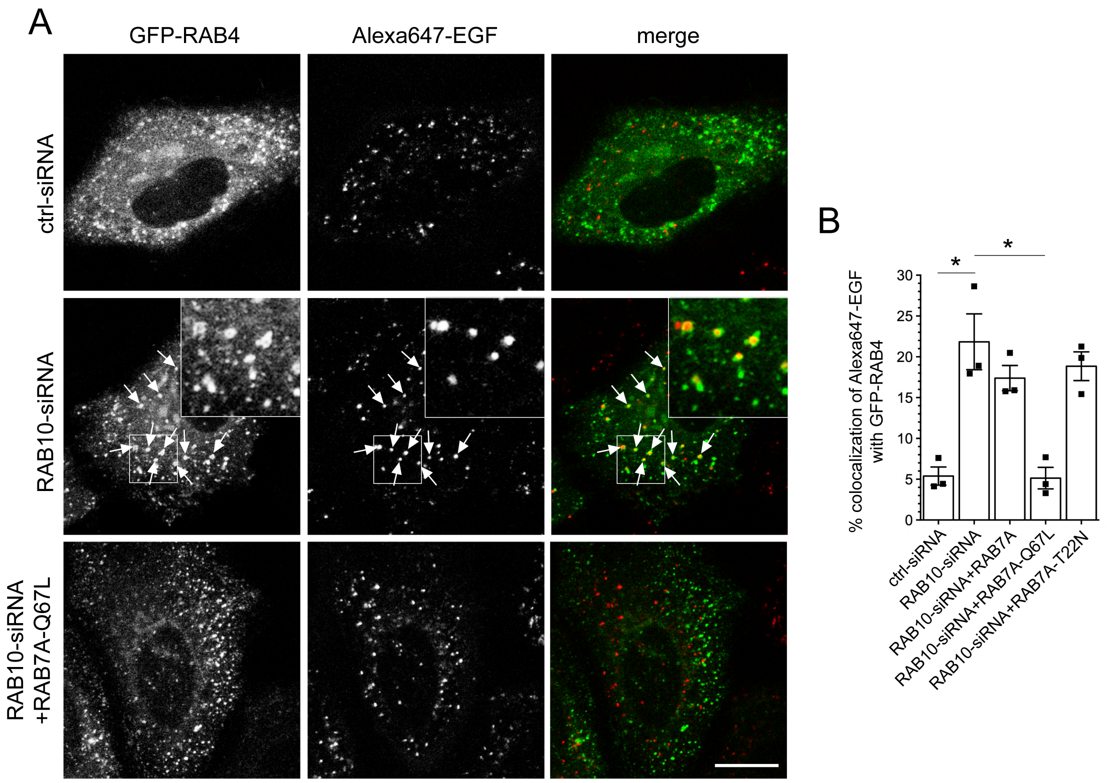

Supplementary Figure 3. Knockdown of RAB10 causes accumulation of EGF in a RAB4-positive compartment rescued upon active RAB7A expression. (**A**) Example of HeLa cells cotransfected with GFP-RAB4 and either ctrl-siRNA or RAB10-siRNA with or without RAB7A-Q67L expression as indicated. Live pictures were taken 20 min upon EGF internalization, and arrows point to GFP-RAB4-positive vesicles containing Alexa647-EGF. An independent picture (543 HeNe laser line) was acquired to confirm coexpression of the distinct mRFP-tagged RAB7A constructs in all cases. Scale bar, 10 μm. (**B**) Quantification of the colocalization of Alexa647-EGF with GFP-RAB4 in either ctrl-siRNA or RAB10-siRNA-treated cells, in the absence or presence of the distinct RAB7A constructs as indicated (Manders´coefficient 1 x 100) from around 20 cells per experiment. N=3 independent experiments; *, $p < 0.05$.

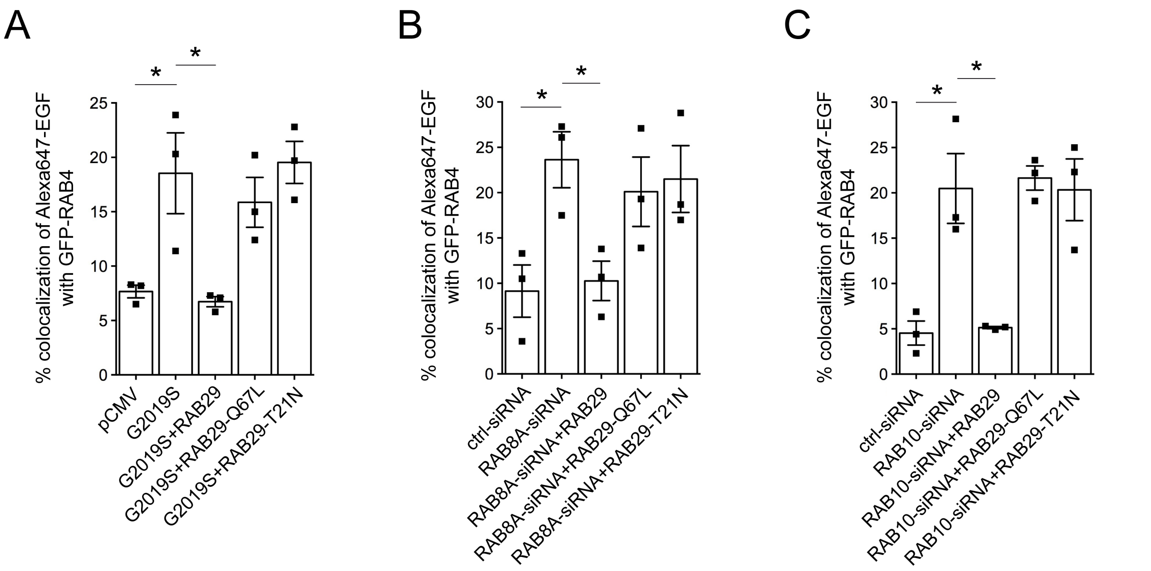

Supplementary Figure 4. RAB29 rescues the accumulation of EGF in a RAB4-positive compartment due to G2019S LRRK2 expression, knockdown of RAB8A or knockdown of RAB10. (**A**) Quantification of the colocalization of Alexa647-EGF with GFP-RAB4 in flag-tagged G2019S LRRK2-expressing cells in either the absence or presence of mRFP-tagged RAB29 constructs as indicated (Manders´coefficient 1 x 100) from around 20 cells per experiment. N=3 independent experiments; *, $p < 0.05$. (**B**) Quantification of the colocalization of Alexa647-EGF with GFP-RAB4 in cells treated with ctrl-siRNA or RAB8A-siRNA in either the absense or presence of mRFP-tagged RAB29 constructs as indicated (Manders´coefficient 1 x 100) from around 20 cells per experiment. N=3 independent experiments; *, $p < 0.05$. (**C**) Quantification of the colocalization of Alexa647-EGF with GFP-RAB4 in cells treated with ctrl-siRNA or RAB10-siRNA in either the absense or presence of mRFP-tagged RAB29 constructs as indicated (Manders´coefficient 1 x 100) from around 20 cells per experiment. N=3 independent experiments; *, $p < 0.05$.

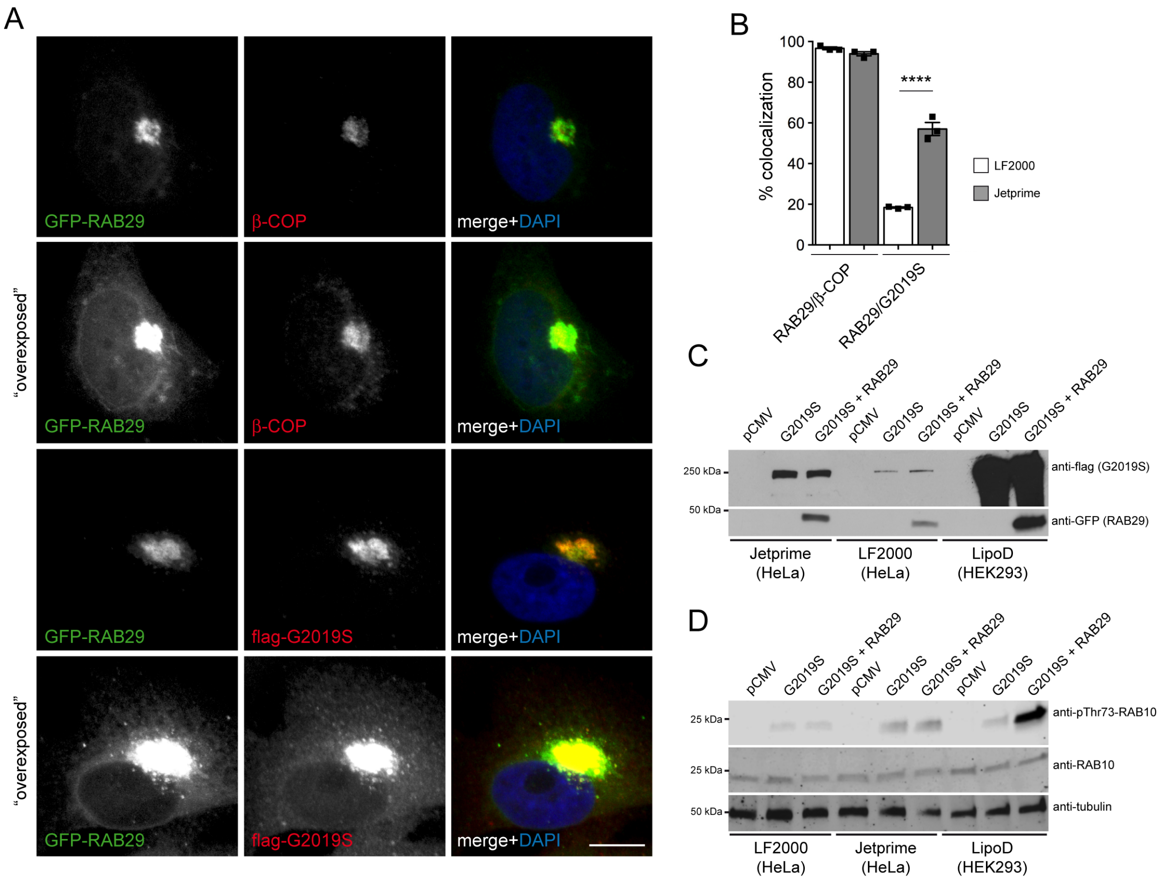

Supplementary Figure 5. RAB29 recruits G2019S LRRK2 to the Golgi complex upon higher expression levels. (**A**) Top: Example of HeLa cell transfected with GFP-tagged RAB29 using Jetprime, and stained with Golgi marker (β-COP) and DAPI. "Overexposed": same cell acquired using confocal settings employed for Lipofectamine 2000 (LF2000)-transfected cells (see Fig. 5A). Bottom: Example of HeLa cell co-transfected with GFP-tagged RAB29 and flag-tagged G2019S LRRK2 using Jetprime, and stained with flag antibody and DAPI. "Overexposed": same cell acquired using confocal settings employed for LF2000-transfected cells (see Fig. 5A). Scale bar, 10 μm. (**B**) Quantification of colocalization of RAB29 with the Golgi marker β-COP, or colocalization of G2019S LRRK2 with RAB29, in cells cotransfected with flag-tagged G2019S LRRK2 and GFP-tagged RAB29 using either LF2000 or Jetprime. One hundred

random transfected cells were scored for colocalization per experiment (N=3 independent experiments). ****, p < 0.001. (**C**) HeLa or HEK293 cells were transfected with either empty pCMV, or co-transfected with flag-tagged G2019S LRRK2 and either GFP or GFP-tagged RAB29 using the indicated lipofection reagents, and extracts (30 μg) analyzed for flag-tagged LRRK2 and GFP-RAB29 expression levels as indicated. (**D**) Cell extracts were analyzed for endogenous phospho-RAB10 levels, total RAB10 levels and tubulin as loading control.

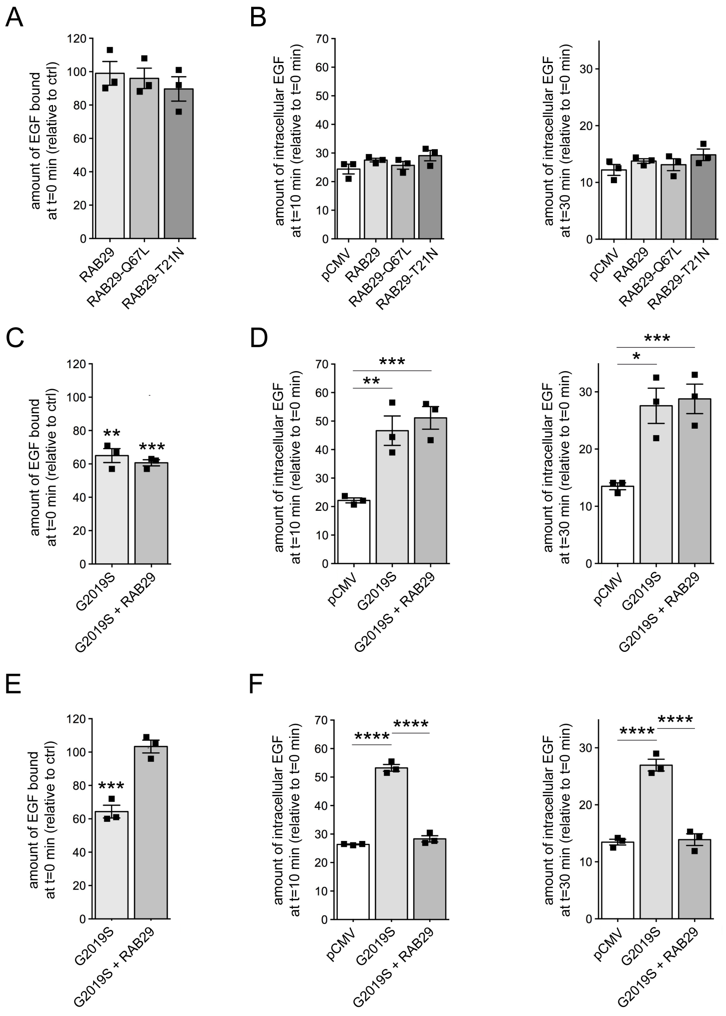

Supplementary Figure 6. Rescue of G2019S LRRK2-mediated EGF trafficking deficits by RAB29 is impaired upon higher expression levels. (**A**) HeLa cells were transfected with pCMV or with the indicated GFP-tagged RAB29 constructs using Jetprime, and surface-bound fluorescent EGF quantified. N=3 independent experiments. (**B**) HeLa cells were transfected with pCMV or with the indicated GFP-tagged RAB29 constructs using Jetprime, and internalized fluorescent EGF was quantified at 10 min (left) and 30 min (right) upon internalization. N=3 independent experiments. (**C**) In parallel experiments, HeLa cells were transfected with pCMV, or co-transfected with flag-tagged G2019S LRRK2 and either GFP or GFP-tagged RAB29 using Jetprime, and

surface-bound fluorescent EGF quantified. N=3 independent experiments. **, $p < 0.01$; ***, $p < 0.005$. (**D**) HeLa cells were transfected with pCMV, or co-transfected with flag-tagged G2019S LRRK2 and either GFP or GFP-tagged RAB29 using Jetprime, and internalized fluorescent EGF was quantified at 10 min (left) and 30 min (right) upon internalization. N=3 independent experiments. *, $p < 0.05$; **, $p < 0.01$; ***, $p < 0.005$. (**E**) In parallel experiments, HeLa cells were transfected with pCMV, or co-transfected with flag-tagged G2019S LRRK2 and either GFP or GFP-tagged RAB29 using LF2000, and surface-bound fluorescent EGF quantified. N=3 independent experiments. ***, $p < 0.005$. (**F**) HeLa cells were transfected with pCMV, or co-transfected with flag-tagged G2019S LRRK2 and either GFP or GFP-tagged RAB29 using LF2000, and internalized fluorescent EGF was quantified at 10 min (left) and 30 min (right) upon internalization. N=3 independent experiments. ****, $p < 0.001$.

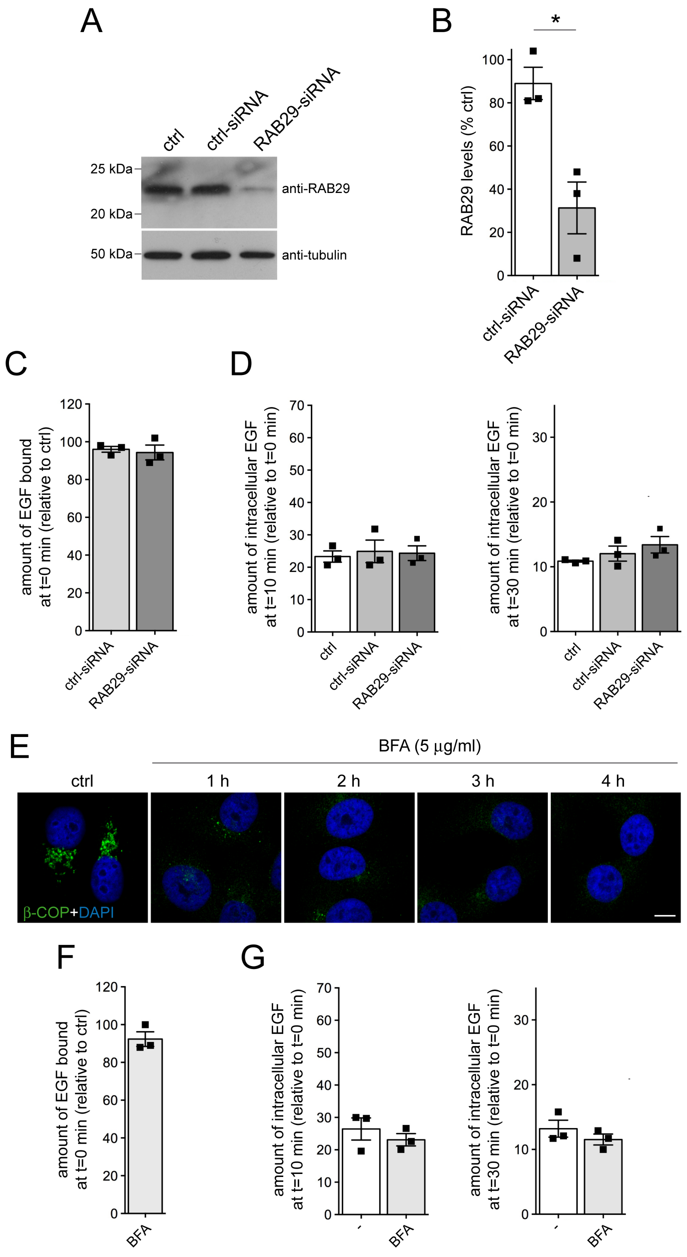

Supplementary Figure 7. siRNA-mediated knockdown of RAB29 or transient disruption of Golgi complex integrity by BFA does not affect EGF trafficking.

(**A**) HeLa cells were either left untreated, transfected with ctrl-siRNA or RAB29-siRNA as indicated, and cell extracts were analyzed by Western blotting for RAB29 levels and tubulin as loading control. (**B**) Quantification of the levels of RAB29 in the presence of ctrl-siRNA or RAB29-siRNA, normalized to the levels in untreated cells. Bars represent

mean ± s.e.m. (N=3 independent experiments; *, $p < 0.05$). (**C**) Cells were either left untreated (ctrl), or treated with ctrl-siRNA or RAB29-siRNA, and the amount of surface-bound fluorescent EGF quantified. N=3 independent experiments. (**D**) Cells were either untreated (ctrl), or treated with ctrl-siRNA or RAB29-siRNA, and internalized fluorescent EGF was quantified at 10 min (left) and 30 min (right). N=3 independent experiments. (**E**) Example of HeLa cells either treated with vehicle (ctrl) or with brefeldin A (BFA) (5 μg/ml) for the indicated amounts of time, followed by staining with a Golgi marker (β-COP) and DAPI. Scale bar, 10 μm. (**F**) Hela cells were either left untreated, or treated for 2 h with BFA as indicated, and the amount of surface-bound fluorescent EGF quantified. N=3 independent experiments. (**G**) Hela cells were either left untreated, or treated for 2 h with BFA as indicated, and internalized fluorescent EGF was quantified at 10 min (left) and 30 min (right) upon internalization. N=3 independent experiments.

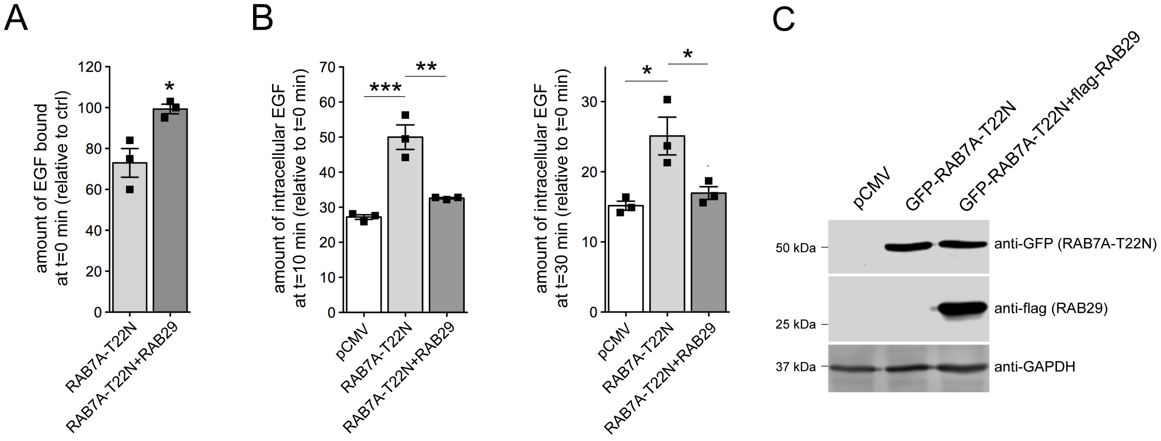

Supplementary Figure 8. The EGF trafficking deficits mediated by dominant-negative RAB7A are reversed upon RAB29 expression. (**A**) HeLa cells were transfected with either empty pCMV vector, or dominant-negative GFP-tagged RAB7A (RAB7A-T22N) in the presence or absence of flag-tagged RAB29, and surface-bound fluorescent EGF was quantified. N=3 independent experiments. *, $p < 0.05$. (**B**) HeLa cells were transfected with the indicated constructs, followed by quantification of internalized fluorescent EGF at 10 (left) and 30 min (right). N=3 independent experiments. *, $p < 0.05$; **, $p < 0.01$; ***, $p < 0.005$. (**C**) HeLa cells were transfected with the indicated constructs, and cell extracts (30 μg) were analyzed by Western blotting for GFP-tagged RAB7A-T22N, flag-tagged RAB29, and GAPDH as loading control.
